# Supplementary material for: Computing molecular excited states on a D-Wave quantum annealer
Source: Sci Rep. 2021 Sep 22;11:18796. doi: 10.1038/s41598-021-98331-y (PMC8458378; doi:10.1038/s41598-021-98331-y)
Supplement: Supplementary file 1 — Supplementary Information. [file 41598_2021_98331_MOESM1_ESM.pdf]

Supplementary Materials
Computing molecular excited states on a D-Wave quantum annealer

Alexander Teplukhin[1], Brian K. Kendrick[1], Susan M. Mniszewski[2], Yu Zhang[1], Ashutosh Kumar[1], Christian F. A. Negre[1], Petr M. Anisimov[3], Sergei Tretiak[1†], and Pavel A. Dub[4*]

[1]Theoretical Division, Los Alamos National Laboratory, Los Alamos, NM 87545, USA
[2]Computer, Computational and Statistical Sciences Division, Los Alamos National Laboratory, Los Alamos, NM 87545, USA
[3]Accelerator Operations and Technology Division, Los Alamos National Laboratory, Los Alamos, NM 87545, USA
[4]Chemistry Division, Los Alamos National Laboratory, Los Alamos, NM 87545, USA
[†]Email: serg@lanl.gov
[*]Corresponding author. Email: pdub@lanl.gov

Contents. This document contains additional results to support the findings of the main text. There are four parts in this documents:

- Part 1 contains the results obtained using the TDHF (CIS) and the QAE running on the D-Wave 2000Q.

Fig. S1. Convergence of CIS excitation energies for the H_2 molecule with respect to basis set.

Fig. S2. CIS excitation energies for the umbrella inversion of ammonia (NH_3).

Table S1. Singlet CIS excitation energies (eV).

Table S2. Triplet CIS excitation energies (eV).

Table S3. Singlet CIS transition dipole moments (au).

Table S4. Singlet CIS oscillator strengths (unitless).

- Part 2 repeats all TDDFT and CIS calculations, but in purely classical mode, where all sub-QUBOs are solved on a CPU using the classical Tabu search technique. No quantum annealer is used.

Fig. S3. Classical convergence of TDDFT excitation energies for the H_2 molecule with respect to basis set.

Fig. S4. Classical convergence of CIS excitation energies for the H_2 molecule with respect to basis set.

Fig. S5. Classical TDDFT excitation energies for the umbrella inversion of ammonia (NH_3).

Fig. S6. Classical CIS excitation energies for the umbrella inversion of ammonia (NH_3).

Table S5. Classical singlet TDDFT excitation energies (eV).

Table S6. Classical triplet TDDFT excitation energies (eV).

Table S7. Classical singlet TDDFT transition dipole moments (au).

Table S8. Classical singlet TDDFT oscillator strengths (unitless).

Table S9. Classical singlet CIS excitation energies (eV).

Table S10. Classical triplet CIS excitation energies (eV).

Table S11. Classical singlet CIS transition dipole moments (au).

Table S12. Classical singlet CIS oscillator strengths (unitless).

- Part 3 demonstrates the heuristic nature of the qbsolv on the example of C_2H_6 in the STO-3G basis set. The computed CIS excitation energies are shown to change from run to run. This is a purely classical calculation on a CPU as well.

Table S13. Classical singlet CIS/STO-3G excitation energies (eV) for C_2H_6, multiple attempts.

Table S14. Classical triplet CIS/STO-3G excitation energies (eV) for C_2H_6, multiple attempts.

- Part 4 shows matrix element distributions for CIS and TDDFT

Fig. S7. Distribution of matrix elements for HOCl computed with 6-31G basis set.

Fig. S8. Distribution of matrix elements for CH_2Cl_2 computed with 6-31G basis set.

Part 1. TDHF (CIS) results obtained on D-Wave 2000Q

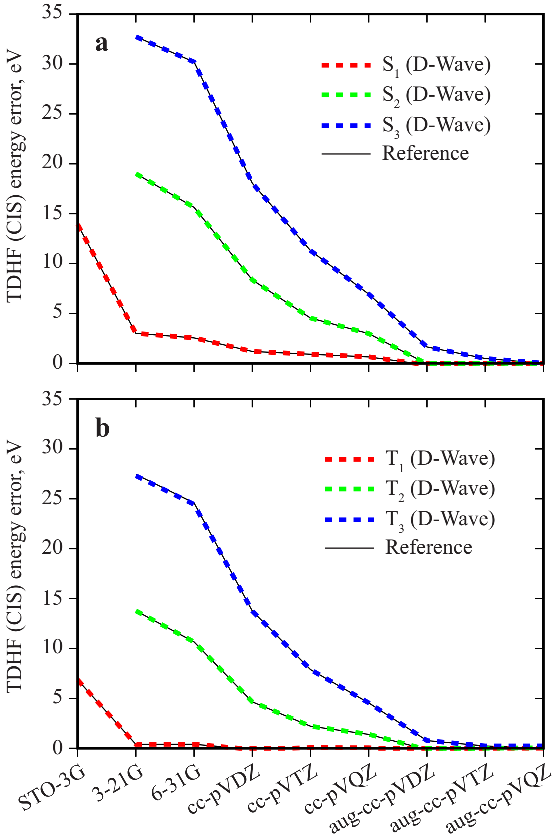

Figure S1: Convergence of CIS excitation energies for the H_2 molecule with respect to basis set. Singlet (a) and triplet (b) calculations are shown separately. The first three excitation energies are computed using PySCF with the QAE and D-Wave 2000Q (dashed red, green and blue curves) and using the unmodified PySCF (thin black curves). The energy error is given relative to the aug-cc-pVQZ reference calculation.

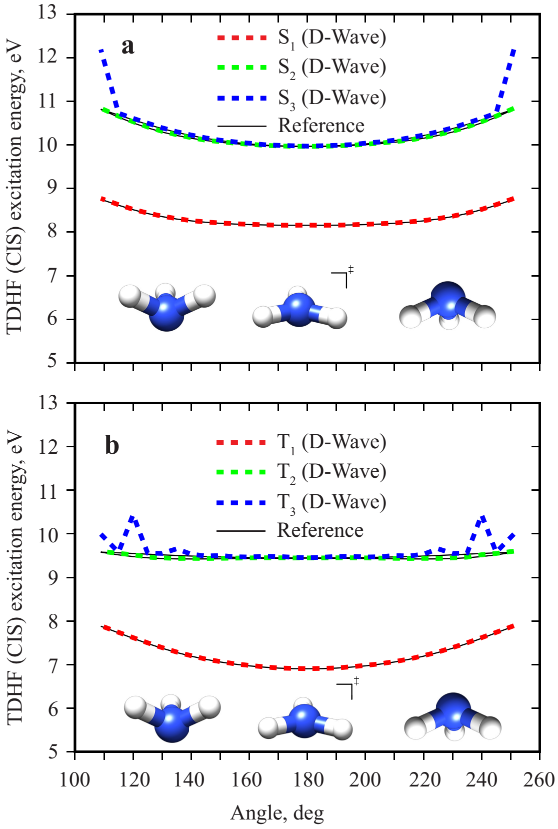

Figure S2: CIS excitation energies for the umbrella inversion of ammonia (NH_3). Singlet (a) and triplet (b) calculations are shown separately. The first three excitation energies are computed using the QAE and D-Wave 2000Q quantum annealer (dashed red, green and blue curves). The reference calculation is shown for comparison (thin black curves). The angle improper is the H-N-H-H dihedral angle.

Table S1: Singlet CIS excitation energies (eV).

Molecule	Mat. size	Reference*			QAE (D-Wave)†			Error‡		
		S_1	S_2	S_3	S_1	S_2	S_3	S_1	S_2	S_3
H_2	3	15.355	28.722	44.273	15.355	28.722	44.268	0.000	0.000	-0.005
H_3^+	5	20.566	20.566	32.503	20.566	20.566	32.503	0.000	0.000	0.000
HF	30	11.844	11.844	16.807	11.845	11.856	16.902	0.002	0.012	0.096
BeH_2	30	7.266	7.266	9.547	7.266	7.268	9.550	0.000	0.001	0.003
H_2O	40	9.497	11.508	11.578	9.502	11.512	11.639	0.005	0.004	0.061
NH_3	50	8.455	10.383	10.383	8.459	10.390	10.405	0.005	0.007	0.022
H_2S	72	7.162	8.059	11.050	7.163	8.061	11.169	0.001	0.002	0.119
HOCl	143	4.112	5.454	7.478	4.261	5.567	11.121	0.149	0.114	3.643
C_2H_6	189	12.641	12.641	13.364	12.710	12.833	13.427	0.070	0.193	0.062
CH_2Cl_2	378	7.756	7.774	7.990	7.938	8.490	11.304	0.182	0.716	3.314

* Reference calculation on CPU using unmodified PySCF
† PySCF was modified to use the QAE and D-Wave 2000Q
‡ Difference between the two types of calculation

Table S2: Triplet CIS excitation energies (eV).

Molecule	Mat. size	Reference*			QAE (D-Wave)†			Error‡		
		T_1	T_2	T_3	T_1	T_2	T_3	T_1	T_2	T_3
H_2	3	10.486	22.741	37.296	10.486	22.741	37.129	0.000	0.000	-0.166
H_3^+	5	15.188	15.188	28.787	15.188	15.188	28.787	0.000	0.000	0.000
HF	30	10.955	10.955	13.067	10.958	11.058	13.133	0.003	0.103	0.066
BeH_2	30	6.570	6.570	7.386	6.570	6.572	7.386	0.000	0.002	0.000
H_2O	40	8.525	10.107	10.892	8.529	10.153	10.913	0.004	0.046	0.021
NH_3	50	7.401	9.714	9.714	7.412	9.724	9.733	0.011	0.010	0.019
H_2S	72	6.512	6.986	8.169	6.519	6.993	8.458	0.008	0.007	0.289
HOCl	143	2.856	3.833	5.337	2.984	4.334	8.717	0.127	0.501	3.379
C_2H_6	189	11.110	11.110	11.365	11.442	12.305	13.507	0.332	1.195	2.142
CH_2Cl_2	378	6.777	6.865	7.072	7.060	8.448	9.986	0.284	1.583	2.915

* Reference calculation on CPU using unmodified PySCF
† PySCF was modified to use the QAE and D-Wave 2000Q
‡ Difference between the two types of calculation

Table S3: Singlet CIS transition dipole moments (au).

Molecule	Mat. size	Reference*			QAE (D-Wave)†			Error‡		
		S_1	S_2	S_3	S_1	S_2	S_3	S_1	S_2	S_3
H_2	3	2.028	0.000	0.104	2.028	0.000	0.117	0.000	0.000	0.013
H_3^+	5	1.490	1.490	0.000	1.491	1.490	0.000	0.000	0.000	0.000
HF	30	0.037	0.037	1.007	0.038	0.038	0.965	0.001	0.001	-0.043
BeH_2	30	0.000	0.000	2.294	0.000	0.000	2.293	0.000	0.000	0.000
H_2O	40	0.065	0.000	0.368	0.065	0.000	0.397	0.000	0.000	0.029
NH_3	50	0.073	0.053	0.053	0.074	0.057	0.066	0.001	0.004	0.013
H_2S	72	0.000	0.012	0.958	0.000	0.011	1.407	0.000	-0.001	0.449
HOCl	143	0.006	0.072	0.027	0.003	0.043	0.013	-0.003	-0.029	-0.013
C_2H_6	189	0.000	0.000	0.000	0.000	0.000	0.000	0.000	0.000	0.000
CH_2Cl_2	378	0.011	0.093	0.000	0.030	0.005	0.259	0.019	-0.088	0.259

* Reference calculation on CPU using unmodified PySCF
† PySCF was modified to use the QAE and D-Wave 2000Q
‡ Difference between the two types of calculation

Table S4: Singlet CIS oscillator strengths (unitless).

Molecule	Mat. size	Reference*			QAE (D-Wave)†			Error‡		
		S_1	S_2	S_3	S_1	S_2	S_3	S_1	S_2	S_3
H_2	3	0.763	0.000	0.113	0.763	0.000	0.127	0.000	0.000	0.014
H_3^+	5	0.751	0.751	0.000	0.751	0.751	0.000	0.000	0.000	0.000
HF	30	0.011	0.011	0.415	0.011	0.011	0.399	0.000	0.000	-0.015
BeH_2	30	0.000	0.000	0.537	0.000	0.000	0.537	0.000	0.000	0.000
H_2O	40	0.015	0.000	0.104	0.015	0.000	0.113	0.000	0.000	0.009
NH_3	50	0.015	0.014	0.014	0.015	0.015	0.017	0.000	0.001	0.003
H_2S	72	0.000	0.002	0.259	0.000	0.002	0.385	0.000	0.000	0.126
HOCl	143	0.001	0.010	0.005	0.000	0.006	0.004	0.000	-0.004	-0.001
C_2H_6	189	0.000	0.000	0.000	0.000	0.000	0.000	0.000	0.000	0.000
CH_2Cl_2	378	0.002	0.018	0.000	0.006	0.001	0.072	0.004	-0.017	0.072

* Reference calculation on CPU using unmodified PySCF
† PySCF was modified to use the QAE and D-Wave 2000Q
‡ Difference between the two types of calculation

Part 2. Classical results using Tabu search to solve subQUBOs

(CPU only, no D-Wave quantum annealer used)

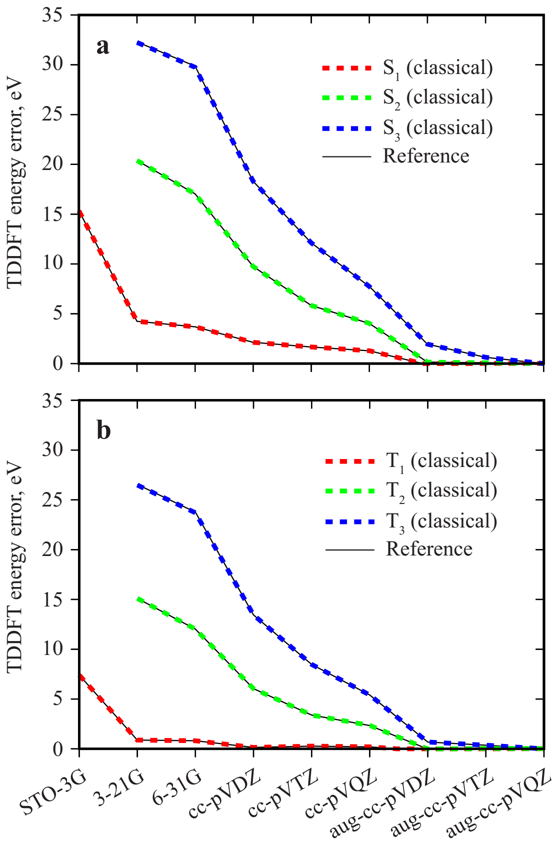

Figure S3: Classical convergence of TDDFT excitation energies for the H_2 molecule with respect to basis set. Singlet (a) and triplet (b) calculations are shown separately. The first three excitation energies are computed using PySCF with the QAE and classical Tabu search (dashed red, green and blue curves) and using the unmodified PySCF (thin black curves). The energy error is given relative to the aug-cc-pVQZ reference calculation.

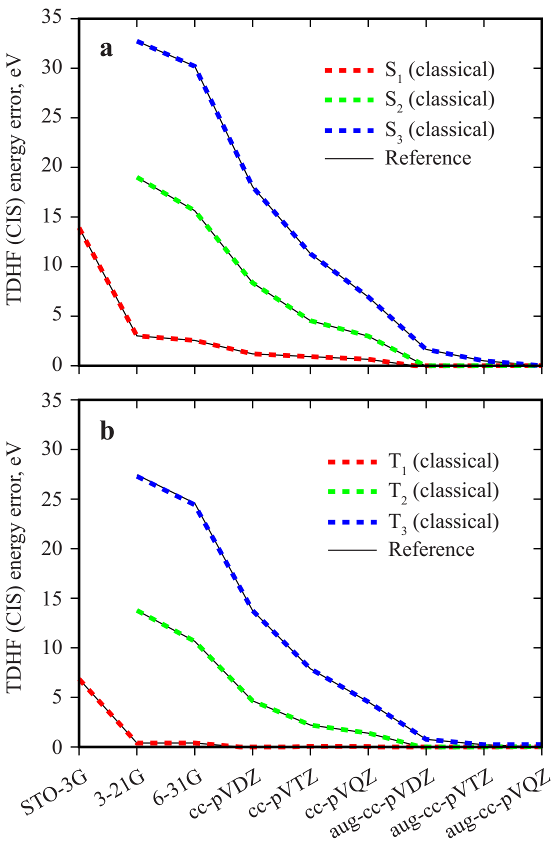

Figure S4: Classical convergence of CIS excitation energies for the H_2 molecule with respect to basis set. Singlet (a) and triplet (b) calculations are shown separately. The first three excitation energies are computed using PySCF with the QAE and classical Tabu search (dashed red, green and blue curves) and using the unmodified PySCF (thin black curves). The energy error is given relative to the aug-cc-pVQZ reference calculation.

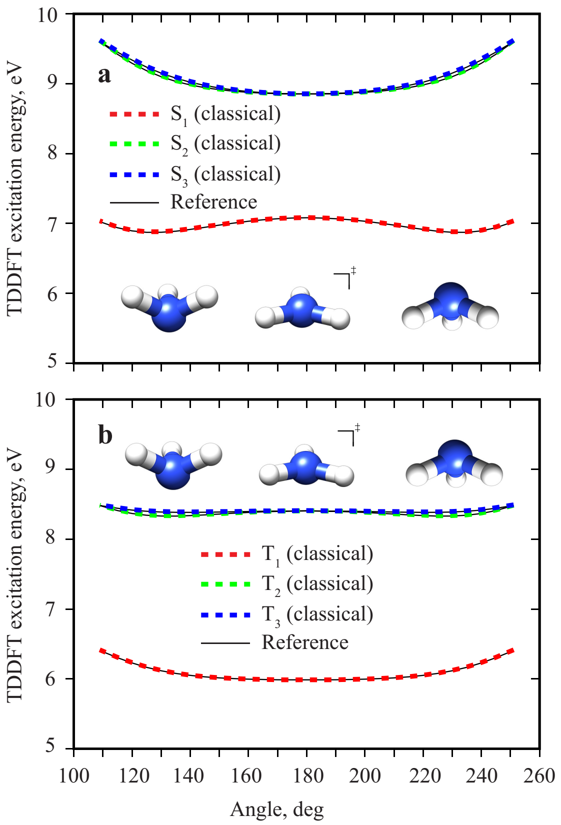

Figure S5: Classical TDDFT excitation energies for the umbrella inversion of ammonia (NH_3). Singlet (a) and triplet (b) calculations are shown separately. The first three excitation energies are computed using the QAE and classical Tabu search (dashed red, green and blue curves). The reference calculation is shown for comparison (thin black curves). The angle is the improper H-N-H-H dihedral angle.

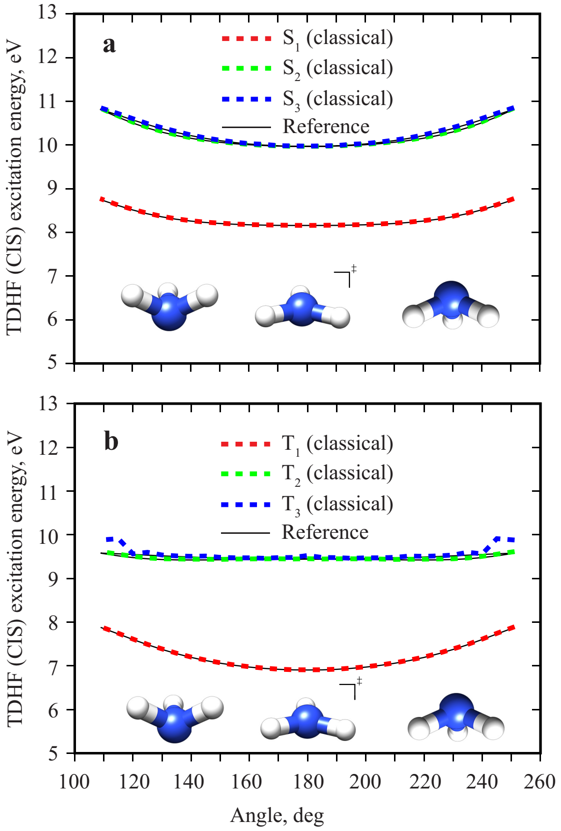

Figure S6: Classical CIS excitation energies for the umbrella inversion of ammonia (NH_3). Singlet (a) and triplet (b) calculations are shown separately. The first three excitation energies are computed using the QAE and classical Tabu search (dashed red, green and blue curves). The reference calculation is shown for comparison (thin black curves). The angle is the improper H-N-H-H dihedral angle.

Table S5: Classical singlet TDDFT excitation energies (eV).

Molecule	Mat. size	Reference*			QAE (classical)†			Error‡		
		S_1	S_2	S_3	S_1	S_2	S_3	S_1	S_2	S_3
H_2	3	15.157	28.645	43.090	15.157	28.645	42.982	0.000	0.000	-0.108
H_3^+	5	20.446	20.446	32.241	20.446	20.446	32.241	0.000	0.000	0.000
HF	30	9.857	9.857	15.405	9.859	9.864	15.446	0.002	0.007	0.041
BeH_2	30	6.420	6.420	8.422	6.420	6.421	8.422	0.000	0.001	0.000
H_2O	40	7.896	9.649	10.044	7.900	9.663	10.049	0.004	0.014	0.005
NH_3	50	7.140	9.186	9.186	7.150	9.195	9.200	0.010	0.009	0.014
H_2S	72	6.617	7.221	9.939	6.618	7.235	9.939	0.001	0.014	0.001
HOCl	143	3.472	4.708	6.321	3.482	4.732	6.345	0.010	0.024	0.024
C_2H_6	189	10.989	10.989	11.571	10.999	10.999	11.604	0.010	0.010	0.033
CH_2Cl_2	378	6.483	6.706	6.854	6.497	6.855	7.082	0.014	0.149	0.228

* Reference calculation on CPU using unmodified PySCF
† PySCF was modified to use the QAE and classical Tabu search to solve subQUBOs
‡ Difference between the two types of calculation

Table S6: Classical triplet TDDFT excitation energies (eV).

Molecule	Mat. size	Reference*			QAE (classical)†			Error‡		
		S_1	S_2	S_3	S_1	S_2	S_3	S_1	S_2	S_3
H_2	3	10.978	23.129	36.199	10.978	23.129	36.131	0.000	0.000	-0.068
H_3^+	5	15.532	15.532	28.900	15.532	15.532	28.900	0.000	0.000	0.000
HF	30	9.104	9.104	12.250	9.110	9.113	12.257	0.005	0.009	0.007
BeH_2	30	5.872	5.872	6.566	5.872	5.874	6.566	0.000	0.002	0.000
H_2O	40	7.101	8.555	9.510	7.104	8.564	9.510	0.003	0.009	0.000
NH_3	50	6.278	8.600	8.600	6.280	8.602	8.605	0.002	0.002	0.005
H_2S	72	6.058	6.312	8.047	6.076	6.312	8.078	0.018	0.000	0.030
HOCl	143	2.356	3.516	5.358	2.372	3.546	5.411	0.016	0.030	0.053
C_2H_6	189	10.350	10.350	10.844	10.396	10.530	10.963	0.046	0.179	0.119
CH_2Cl_2	378	5.840	5.853	6.174	5.859	6.187	6.387	0.019	0.333	0.214

* Reference calculation on CPU using unmodified PySCF
† PySCF was modified to use the QAE and classical Tabu search to solve subQUBOs
‡ Difference between the two types of calculation

Table S7: Classical singlet TDDFT transition dipole moments (au).

Molecule	Mat. size	Reference*			QAE (classical)†			Error‡		
		S_1	S_2	S_3	S_1	S_2	S_3	S_1	S_2	S_3
H_2	3	1.974	0.000	0.133	1.974	0.000	0.204	0.000	0.000	0.071
H_3^+	5	1.453	1.453	0.000	1.453	1.453	0.000	0.000	0.000	0.000
HF	30	0.038	0.038	1.030	0.038	0.039	1.075	0.000	0.001	0.045
BeH_2	30	0.000	0.000	2.447	0.000	0.000	2.447	0.000	0.000	0.000
H_2O	40	0.062	0.374	0.000	0.062	0.359	0.000	0.001	-0.016	0.000
NH_3	50	0.058	0.051	0.051	0.054	0.052	0.060	-0.003	0.001	0.010
H_2S	72	0.000	0.016	0.752	0.000	0.015	0.747	0.000	-0.001	-0.004
HOCl	143	0.004	0.037	0.024	0.003	0.029	0.031	-0.001	-0.008	0.006
C_2H_6	189	0.000	0.000	0.000	0.000	0.000	0.000	0.000	0.000	0.000
CH_2Cl_2	378	0.009	0.221	0.000	0.011	0.017	0.000	0.002	-0.204	0.000

* Reference calculation on CPU using unmodified PySCF

† PySCF was modified to use the QAE and classical Tabu search to solve subQUBOs

‡ Difference between the two types of calculation

Table S8: Classical singlet TDDFT oscillator strengths (unitless).

Molecule	Mat. size	Reference*			QAE (classical)†			Error‡		
		S_1	S_2	S_3	S_1	S_2	S_3	S_1	S_2	S_3
H_2	3	0.733	0.000	0.141	0.733	0.000	0.215	0.000	0.000	0.074
H_3^+	5	0.728	0.728	0.000	0.728	0.728	0.000	0.000	0.000	0.000
HF	30	0.009	0.009	0.389	0.009	0.009	0.407	0.000	0.000	0.018
BeH_2	30	0.000	0.000	0.505	0.000	0.000	0.505	0.000	0.000	0.000
H_2O	40	0.012	0.089	0.000	0.012	0.085	0.000	0.000	-0.003	0.000
NH_3	50	0.010	0.011	0.011	0.010	0.012	0.014	-0.001	0.000	0.002
H_2S	72	0.000	0.003	0.183	0.000	0.003	0.182	0.000	0.000	-0.001
HOCl	143	0.000	0.004	0.004	0.000	0.003	0.005	0.000	-0.001	0.001
C_2H_6	189	0.000	0.000	0.000	0.000	0.000	0.000	0.000	0.000	0.000
CH_2Cl_2	378	0.002	0.036	0.000	0.002	0.003	0.000	0.000	-0.033	0.000

* Reference calculation on CPU using unmodified PySCF

† PySCF was modified to use the QAE and classical Tabu search to solve subQUBOs

‡ Difference between the two types of calculation

Table S9: Classical singlet CIS excitation energies (eV).

Molecule	Mat. size	Reference*			QAE (classical)†			Error‡		
		S_1	S_2	S_3	S_1	S_2	S_3	S_1	S_2	S_3
H_2	3	15.355	28.722	44.273	15.355	28.722	44.264	0.000	0.000	-0.009
H_3^+	5	20.566	20.566	32.503	20.566	20.566	32.503	0.000	0.000	0.000
HF	30	11.844	11.844	16.807	11.845	11.849	16.839	0.001	0.006	0.032
BeH_2	30	7.266	7.266	9.547	7.266	7.268	9.549	0.000	0.002	0.002
H_2O	40	9.497	11.508	11.578	9.502	11.513	11.631	0.005	0.005	0.053
NH_3	50	8.455	10.383	10.383	8.459	10.392	10.405	0.005	0.009	0.022
H_2S	72	7.162	8.059	11.050	7.174	8.061	11.179	0.012	0.002	0.129
HOCl	143	4.112	5.454	7.478	4.248	5.568	10.999	0.136	0.115	3.521
C_2H_6	189	12.641	12.641	13.364	12.706	13.366	14.123	0.065	0.725	0.759
CH_2Cl_2	378	7.756	7.774	7.990	7.941	8.202	10.390	0.186	0.427	2.400

* Reference calculation on CPU using unmodified PySCF

† PySCF was modified to use the QAE and classical Tabu search to solve subQUBOs

‡ Difference between the two types of calculation

Table S10: Classical triplet CIS excitation energies (eV).

Molecule	Mat. size	Reference*			QAE (classical)†			Error‡		
		S_1	S_2	S_3	S_1	S_2	S_3	S_1	S_2	S_3
H_2	3	10.486	22.741	37.296	10.486	22.741	37.144	0.000	0.000	-0.151
H_3^+	5	15.188	15.188	28.787	15.188	15.188	28.787	0.000	0.000	0.000
HF	30	10.955	10.955	13.067	10.961	11.061	13.091	0.006	0.105	0.024
BeH_2	30	6.570	6.570	7.386	6.570	6.573	7.386	0.000	0.003	0.000
H_2O	40	8.525	10.107	10.892	8.530	10.172	11.022	0.005	0.065	0.131
NH_3	50	7.401	9.714	9.714	7.412	9.723	10.264	0.010	0.009	0.550
H_2S	72	6.512	6.986	8.169	6.523	6.987	8.313	0.012	0.001	0.144
HOCl	143	2.856	3.833	5.337	3.019	4.333	8.314	0.163	0.500	2.977
C_2H_6	189	11.110	11.110	11.365	11.498	11.624	12.103	0.388	0.514	0.738
CH_2Cl_2	378	6.777	6.865	7.072	7.016	7.498	9.016	0.239	0.633	1.944

* Reference calculation on CPU using unmodified PySCF

† PySCF was modified to use the QAE and classical Tabu search to solve subQUBOs

‡ Difference between the two types of calculation

Table S11: Classical singlet CIS transition dipole moments (au).

Molecule	Mat. size	Reference*			QAE (classical)†			Error‡		
		S_1	S_2	S_3	S_1	S_2	S_3	S_1	S_2	S_3
H_2	3	2.028	0.000	0.104	2.028	0.000	0.121	0.000	0.000	0.017
H_3^+	5	1.490	1.490	0.000	1.491	1.490	0.000	0.000	0.000	0.000
HF	30	0.037	0.037	1.007	0.038	0.038	1.007	0.000	0.001	-0.001
BeH_2	30	0.000	0.000	2.294	0.000	0.000	2.293	0.000	0.000	-0.001
H_2O	40	0.065	0.000	0.368	0.064	0.000	0.396	-0.001	0.000	0.029
NH_3	50	0.073	0.053	0.053	0.075	0.064	0.066	0.002	0.011	0.013
H_2S	72	0.000	0.012	0.958	0.000	0.012	1.166	0.000	0.000	0.208
HOCl	143	0.006	0.072	0.027	0.004	0.044	0.438	-0.002	-0.028	0.411
C_2H_6	189	0.000	0.000	0.000	0.000	0.000	1.020	0.000	0.000	1.020
CH_2Cl_2	378	0.011	0.093	0.000	0.023	0.001	0.212	0.012	-0.092	0.212

* Reference calculation on CPU using unmodified PySCF

† PySCF was modified to use the QAE and classical Tabu search to solve subQUBOs

‡ Difference between the two types of calculation

Table S12: Classical singlet CIS oscillator strengths (unitless).

Molecule	Mat. size	Reference*			QAE (classical)†			Error‡		
		S_1	S_2	S_3	S_1	S_2	S_3	S_1	S_2	S_3
H_2	3	0.763	0.000	0.113	0.763	0.000	0.131	0.000	0.000	0.018
H_3^+	5	0.751	0.751	0.000	0.751	0.751	0.000	0.000	0.000	0.000
HF	30	0.011	0.011	0.415	0.011	0.011	0.415	0.000	0.000	0.001
BeH_2	30	0.000	0.000	0.537	0.000	0.000	0.536	0.000	0.000	0.000
H_2O	40	0.015	0.000	0.104	0.015	0.000	0.113	0.000	0.000	0.009
NH_3	50	0.015	0.014	0.014	0.015	0.016	0.017	0.000	0.003	0.003
H_2S	72	0.000	0.002	0.259	0.000	0.002	0.319	0.000	0.000	0.060
HOCl	143	0.001	0.010	0.005	0.000	0.006	0.118	0.000	-0.004	0.113
C_2H_6	189	0.000	0.000	0.000	0.000	0.000	0.353	0.000	0.000	0.353
CH_2Cl_2	378	0.002	0.018	0.000	0.005	0.000	0.054	0.002	-0.018	0.054

* Reference calculation on CPU using unmodified PySCF

† PySCF was modified to use the QAE and classical Tabu search to solve subQUBOs

‡ Difference between the two types of calculation

Part 3. Multiple attempts (runs) to demonstrate the heuristic nature of qbsolv

(CPU only, no D-Wave quantum annealer used)

Table S13: Classical singlet CIS/STO-3G excitation energies (eV) for C_2H_6, multiple attempts.

Attempt #	QAE (CPU)†			Error‡		
	S_1	S_2	S_3	S_1	S_2	S_3
1	20.246	20.574	21.080	0.004	0.333	0.266
2	20.246	20.440	20.952	0.004	0.199	0.139
3	20.248	20.382	20.848	0.006	0.140	0.034
4	20.244	20.375	20.955	0.003	0.134	0.141
5	20.248	20.277	20.992	0.006	0.035	0.178
6	20.244	20.253	20.834	0.002	0.011	0.021
7	20.244	20.457	21.189	0.002	0.215	0.375
8	20.246	20.279	21.080	0.004	0.038	0.266
9	20.246	20.355	20.945	0.004	0.114	0.131
10	20.245	20.327	20.989	0.003	0.085	0.175
Ref*	20.242	20.242	20.814	-	-	-

* Reference calculation on CPU using unmodified PySCF

† PySCF was modified to use the QAE and classical Tabu search to solve subQUBOs

‡ Difference between the two types of calculation

Table S14: Classical triplet CIS/STO-3G excitation energies (eV) for C_2H_6, multiple attempts.

Attempt #	QAE (CPU)†			Error‡		
	T_1	T_2	T_3	T_1	T_2	T_3
1	15.420	17.160	18.013	0.036	0.355	1.153
2	15.404	16.811	17.948	0.020	0.005	1.089
3	15.407	17.296	18.006	0.023	0.490	1.147
4	15.419	16.824	17.425	0.035	0.018	0.565
5	15.407	17.333	18.713	0.023	0.528	1.854
6	15.421	17.036	18.223	0.037	0.230	1.364
7	15.401	17.356	18.248	0.017	0.551	1.388
8	15.395	17.212	18.638	0.011	0.407	1.778
9	15.395	17.262	17.983	0.011	0.457	1.123
10	15.391	17.160	18.644	0.007	0.355	1.784
Ref*	15.384	16.805	16.860	-	-	-

* Reference calculation on CPU using unmodified PySCF

† PySCF was modified to use the QAE and classical Tabu search to solve subQUBOs

‡ Difference between the two types of calculation

Part 4. Matrix element distributions

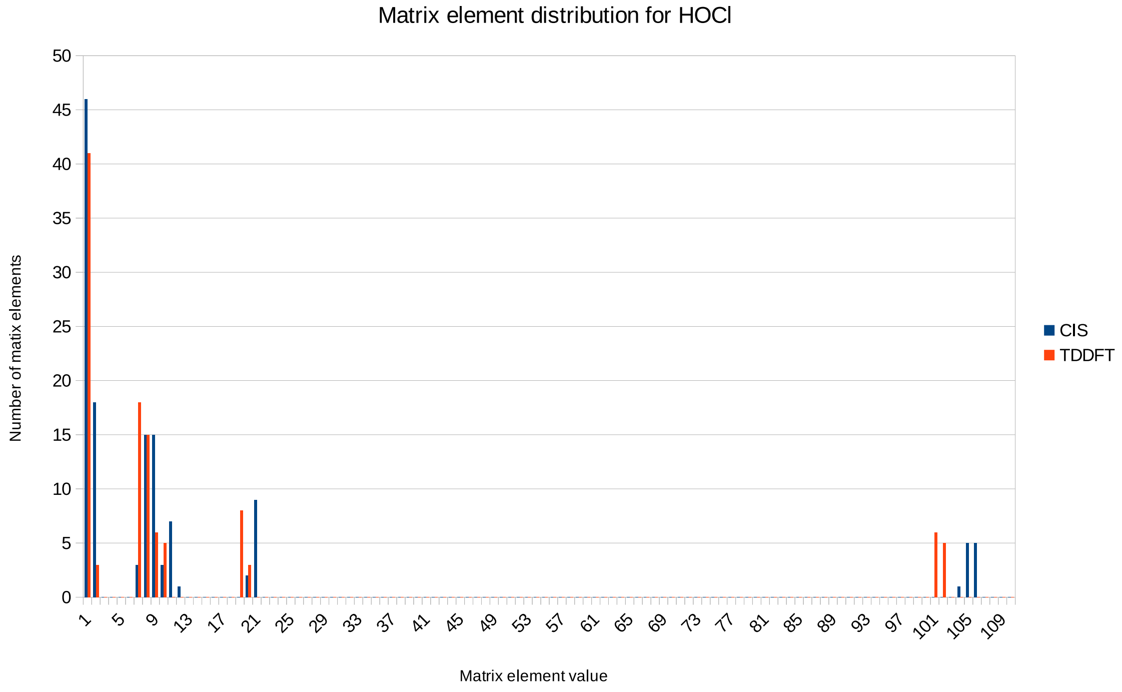

Figure S7: Distribution of matrix elements for HOCl computed with 6-31G basis set. The bin size is one. The largest TDDFT matrix elements (red bars) tend to be slightly smaller than CIS matrix elements (blue bars). Matrix elements less than one are excluded.

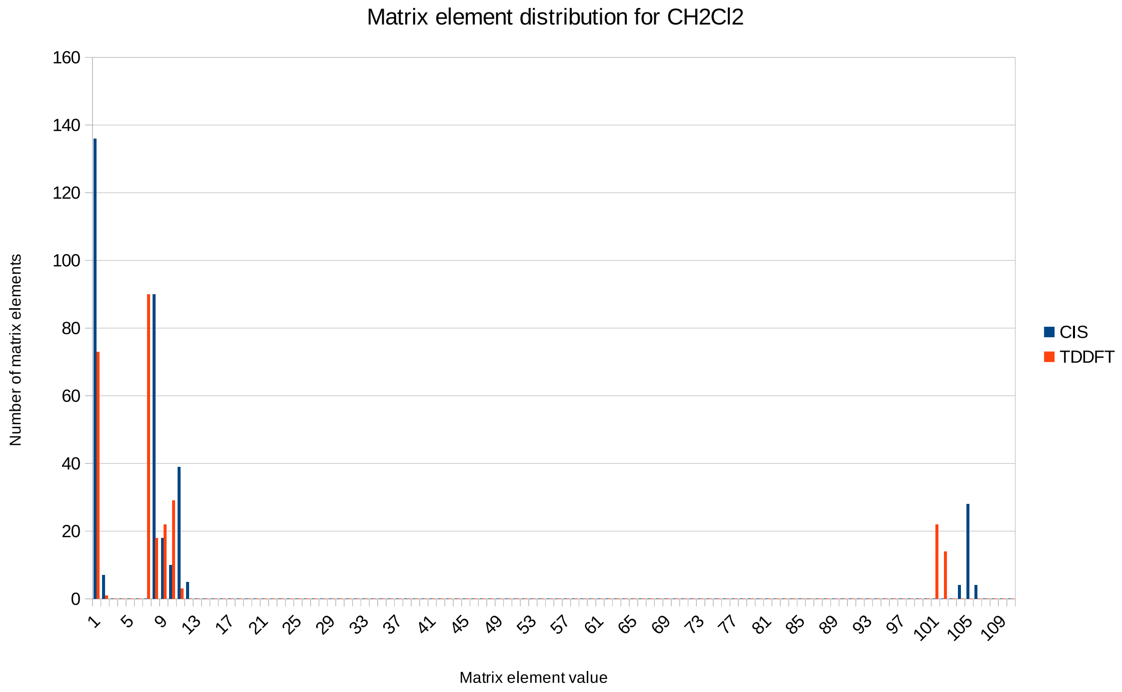

Figure S8: Distribution of matrix elements for CH_2Cl_2 computed with 6-31G basis set. The bin size is one. The largest TDDFT matrix elements (red bars) tend to be slightly smaller than CIS matrix elements (blue bars). Matrix elements less than one are excluded.
